# Influential Factors on the Relative Age Effect in Alpine Ski Racing

**DOI:** 10.1371/journal.pone.0134744

**Published:** 2015-08-07

**Authors:** Lisa Müller, Erich Müller, Carolin Hildebrandt, Elmar Kornexl, Christian Raschner

**Affiliations:** 1 Department of Sport Science, University of Innsbruck, Innsbruck, Tyrol, Austria; 2 Department of Sport Science and Kinesiology, University of Salzburg, Salzburg, Salzburg, Austria; VU University Amsterdam, NETHERLANDS

## Abstract

The relative age effect (RAE), which refers to an over-representation of selected athletes born early in the selection year, was proven to be present in alpine ski racing in all age categories at both national and international levels. However, the influential factors on, or the causal mechanisms of, the RAE are still unknown. Therefore, the aim of the present study was to examine three possible influential factors on the relative age effect in alpine skiing: physical performance, anthropometric characteristics and biological maturational status. The study included the investigation of 282 elite Austrian youth ski racers and 413 non-athletes (comparison group) of the same age (10–13 years) and region. Six physical performance tests were performed, body mass and height were assessed, and the age at peak height velocity (APHV) was calculated. A significant RAE was present in the ski racers. No differences were shown in the physical performance characteristics or in the calculated APHV between the relative age quarters. These results suggest that ski racers born in the last quarter can counteract the relative age disadvantages if they already present the same level of physical performance and maturational status as those born at the beginning of the year. The height and weight of ski racers born at the beginning of the year were significantly higher compared to the non-athletes, and ski racers born in relative age quarter 1 were taller and heavier compared to the ski racers of the other quarters. This indicates that the anthropometric characteristics influence the selection process in alpine ski racing, and that relatively older athletes are more likely to be selected if they exhibit advanced anthropometric characteristics.

## Introduction

In many sports, participants of youth competitions are divided into competition categories based on their chronological age with the goals of guaranteeing fair competition and reducing the effect of developmental differences between athletes [1–3]. Even though this strategy is well intentioned, it is responsible for creating chronological age advantages, since it leads to age differences of up to 12 months between individuals [1,4]. This age difference leads to a phenomenon known as the *relative age effect* (RAE). The RAE was first documented in Canadian ice hockey [5]; since then, its presence has been proven in many other sports as well [1,6,7]. A RAE exists when the relative age quarter distribution of a selected sports group shows a skewed distribution with an over-representation of athletes whose birth months are close to the cut-off date for the competition categories [1,8], even though the relative age quarter distribution of the general population shows an even distribution among the quarters [8,9].

An age difference of up to 12 months can lead to the following advantages for the relatively older athletes: first is the developmental advantage, which leads to a much broader experience in training and competition, and a resulting higher level of performance [1]. Second are the physical and mental advantages, in which the physical advantages in particular are emphasized [1]. The relatively older athletes are often taller and heavier, which is beneficial in various types of sport, as for example in ice hockey [10] and alpine skiing [11]. Accordingly, relatively older athletes within an annual age cohort have significant attainment advantages compared to relatively younger counterparts [12], and as a result, are favorably selected [8]. If the relatively older athletes are also more mature compared to the relatively younger ones, the aforementioned advantages will be even greater [8]; thus, athletes with advanced biological maturity are more likely to be selected due to performance advantages in various fitness parameters [10,13,14].

Furthermore, the athletes selected for elite squads (who are most often relatively older) have better training facilities and higher qualified coaches, whereas the relatively younger ones often are not included in the talent development program; they do not have the same training possibilities, even though they are on average as talented as the relatively older athletes [15]. As a consequence, the advantages of the relatively older athletes would increase once again [1], and many talented young athletes are forfeited because they drop out of a sport early [16]. From an ethical point of view, the presence of a RAE in various types of sport indicates that the talent development systems seem to be biased against relatively younger athletes, which results in fewer opportunities of their reaching the elite level despite their talents and efforts [6].

The RAE also represents a problem in alpine ski racing. A RAE already exists at national youth levels in Austrian [6,17] and Swiss ski racers, aged 7 to 15 years [18]. This effect continues to be present at international youth levels, and was manifest at the 1^st^ Winter Youth Olympic Games (YOG) in Innsbruck in 2012 (ski racers aged 15 to 16 years) [9], the FIS Junior World Ski Championships 2009–2011 (athletes aged 16 to 20 years) [19], as well as at the international elite level, the FIS World Cup [19,20]. In summary, the RAE in alpine ski racing is present in all age categories at both national, as well as at international levels [6]. However, the influential factors responsible for the existence of the RAE in alpine ski racing still remain unclear and have yet to be assessed [6]. It can be hypothesized that talent in a sport does not depend on the birth month [1,15]; consequently, it can be assumed that there is a severe loss of talent as a consequence of the existing RAE in all age categories of alpine ski racing [6]. Wattie et al. [21] mention that a RAE can be better explained using a domain-specific model; this means that the diverse interacting factors that influence the participation should be investigated for each specific sport. The factors influencing the participation and consequently causing a RAE can differ from sport to sport. Consequently, it is necessary to find the most important influential mechanisms on the RAE also in alpine ski racing, which has not been performed before. As a consequence, by knowing the influential mechanisms, strategies in the talent development systems can be changed to minimize the RAE in alpine ski racing.

Alpine skiing is a sport that requires a high level of physical fitness [22–24]; variables such as aerobic capacity, lower limb muscle strength, power and power endurance, among others, have been documented as important factors in alpine ski racing performance [22–26]. Therefore, it can be hypothesized that physical performance level could represent one mechanism influencing the participation rate in alpine ski racing and could consequently be one of the causal mechanisms responsible for the RAE in this sport. The selection process may be based on false assumptions, and as a consequence may be identifying and selecting skillful youngsters according to pre-adult physical disposition and without differentiating skills from advanced maturation [27]. Sandmayr [28] reported that pupils of skiing-specific secondary modern schools showed significantly better results in various fitness tests compared to pupils of sport-specific or normal secondary modern schools. With this effect in mind, it can be assumed that the level of performance of physical motor skills is an essential and crucial component for success in alpine skiing, and may be more important than in other sports. [17] Consequently, although other studies in soccer [29] or rugby [7,27] did not find that physical performance level has a significant influence on the RAE, it should nevertheless be assessed in alpine ski racing.

Alpine skiing is a sport in which athletes with higher body mass and height have advantages. For example, significant correlations could be found between performance (slalom) and anthropometric components [11]. Additionally, since many authors explain the over-representation of athletes born early in the selection year as a result of being chosen for their larger anthropometric dimensions, it might be assumed that anthropometric characteristics could be influential factors of the RAE in alpine ski racing. However, in youth soccer (U10-U19) no significant influences of anthropometric characteristics on the RAE could be assessed [29–31]; the soccer players of the four relative age quarters did not significantly differ in height and weight. In contrast to soccer, Raschner et al. [9] found that body mass and height had a significant influence on the RAE among male participants in the YOG 2012 with athletes of relative age quarter 1 showing higher values compared to those of the other quarters. In that study, athletes of winter sport disciplines were examined; hence, the results lead to the assumption that anthropometric characteristics could be an influential factor of the RAE in alpine ski racing also, which nevertheless should be examined.

Gil et al. [32] examined the influence of maturational status on the RAE among 88 Spanish youth soccer players, and Deprez et al. [12] assessed this influence in 374 Belgian youth soccer players. No significant differences were shown between the players born in the single relative age quarters; maturational status did not significantly differ between the athletes of the four relative age quarters. However, as already mentioned, athletes with advanced biological maturity are more likely to be selected due to performance advantages in various fitness parameters [10,13,14]. Sherar et al. [10] reported that with every 1-month increase in the age at peak height velocity (APHV), which is often used as non-invasive method of assessing biological maturation, adolescent ice hockey players became 17% less likely to be selected for a competitive team. This means that the higher the APHV of an athlete, the less mature he/she is; hence, the results of Sherar et al. [10] indicate that early maturing youths have a selection advantage. Consequently, relatively older athletes who are also more mature have an additional increased likelihood for selection. Because body mass and height are both associated with maturation [11] and because taller and heavier athletes have advantages in alpine ski racing, it could be assumed that biological maturation would be an influential factor of the RAE in alpine ski racing. However, this assumption has to be assessed.

Therefore, the aim of the present study was to assess the potential influential factors on the RAE in alpine ski racing and in this context the influences of 1) the level of physical performance, 2) the anthropometric parameters (body mass and height) and 3) biological maturation. Additionally, a comparison group of non-athletes of the same age and region should be included in order to be able to investigate, whether the potential influential factors are generally valid, or are applicable only to alpine ski racers.

## Methods

### Participants and design

Elite youth ski racers of Austrian ski boarding schools and pupils of secondary modern schools and grammar schools (aged 10–13 years) from the same regions (comparison group of non-athletes) participated in this study. Pupils of the comparison group did not attend a skiing-specific secondary modern school; hence, they were not elite youth ski racers and represented an average population group of 10- to 13-year-old youths. In total, 695 Austrian pupils (328 males, 367 females) were examined: 282 ski racers (155 males, 127 females); the comparison group consisted of 413 pupils (173 males, 240 females). All participants were tested once between May and November, 2014.

### Ethics Statement

Parents and participants were informed of the study aims, requirements and risks before providing written informed consent. The study was performed according to the Declaration of Helsinki. The study was approved by the Institutional Review Board of the Department of Sport Science of the University of Innsbruck and the Board for Ethical Questions in Science of the University of Innsbruck (Nr.: 2/2014).

### Measurements and Procedures

The birth dates were collected and all the participants were categorized into four relative age quarters according to their month of birth. Since the cut-off date for grouping the various competition categories in alpine skiing is the 1^st^ of January, the birth months were split into quarters to calculate relative age as follows: quarter one (Q1) included the months January to March; quarter 2 (Q2), the months April to June; quarter three (Q3), the months July to September; and quarter four (Q4), the months October to December. The relative age quarter distribution of the comparison group of non-athletes was used as expected distribution for the analyses concerning the existence of a RAE within the youth ski racers (separated for gender), because Wattie et al. [21], for example, demand that the actual population distributions need to be used for comparison distributions.

The following anthropometric characteristics were assessed: body height (0.1 cm, Seca Portable Stadiometer, Hamburg, Germany), sitting height (0.1 cm, Seca Portable Stadiometer, Hamburg Germany; sitting height table) and body mass (0.1 kg, Seca, Hamburg, Germany) according to previously described procedures [33].

Estimation of biological maturation of each participant was calculated using the non-invasive method based on the anthropometric measurements mentioned above, the calculation of leg length as the difference between body height and sitting height, and actual chronological age at the time of measurement as proposed by Mirwald et al. [34]. These parameters were used to predict maturity offset (MO) as the time before or after peak height velocity (PHV), which was calculated separately for boys and girls using the prediction equations of Mirwald et al. [34]. The predicted age at PHV (APHV) was calculated as the difference between chronological age (CA) and MO.

After a 15-minute standardized warm-up period, the participants completed a test battery of six physical performance tests (Table 1), as described by Raschner et al. [35,36]. All participants had experience in the performance tests. For this analysis, a smaller sample size of 139 pupils (77 males, 62 females) was utilized, with 53 ski racers (31 males, 22 females), and 86 pupils from the comparison group (46 males, 40 females) participating in the physical performance tests. All other measurements were completed using the whole sample of 695 pupils.

**Table 1:** Description of physical performance tests.

test	skill tested	short description	parameter examined	attempts (break)	ICC (test-retest)
S3-Check-Test	postural stability	subject stood on an uniaxial instable platform (left-right instability) and tried to keep it level; knees were slightly flexed; arms free	stabilization index [index]	2 (15 seconds)	female: 0.90ˇ male: 0.91ˇ
Jump Coordination Test (JCT)	jump agility	course with hurdles of varied heights (15–36 cm); 26 two footed jumps (forwards, backwards, sideways) as quick as possible	time [0.1 s]	3 (3 minutes)	female: 0.89° male: 0.91°
Counter movement jump (CMJ)	explosive leg power / strength	subject started movement standing erect, then quickly bent at the hip, knee and ankles before starting upward motion; hands held on the hips	jumping height [0.1 cm]	3 (30 seconds)	female: 0.96° male: 0.97°
Drop jump (DJ)	reactive strength	subject dropped from a 40 cm high podium; had to jump as high as possible with minimal ground reaction time	reactive strength index [1 mm/1 ms]	3 (30 seconds)	female: 0.97° male: 0.92°
Unilateral leg press strength test (ULST)	max. isometric leg extension strength	with a knee-angle of 100° (180° = fully extended knee), the subject performed three one-leg isometric leg extensions	leg force relative to body mass [0.1 N/kg]	2 (15 seconds)	female: 0.95° male: 0.96°
Modified Back-Check-Test	max. isometric core flexion and extension strength	subject stood in upright standing position, with knees slightly flexed, pelvis stabilized; pads were set at sternum level; athlete contracted maximally against the cushioned pad (flexion and extension)	flexion and extension strength relative to body mass [0.1 N/kg]	3 (15 seconds)	female: 0.94ˇ male: 0.98ˇ

°Raschner et al. [35];

^ˇ^ Raschner et al. [36];

ICC = intraclass correlation coefficient

### Statistical Analysis

To assess the differences between the relative age quarter distribution of the ski racers (observed distribution) and that of the comparison group (expected distribution, nearly equal distribution with approximately 25% born in each quarter), chi^2^-tests (χ^2^) were used for the total sample and for samples separated by gender. The effect size ω for the chi^2^-tests was calculated, as proposed by Wattie et al. [21]. Odds ratios (ORs) and 95% confidence intervals (95% CI) were calculated for relative age quartile distribution for the total sample and according to gender, as proposed by Cobley et al. [2].

Due to the fact that the participants’ ages ranged from 10 to 13 years, a z-transformation (calculated separately by birth year and gender) of the data of the physical performance tests and the anthropometric parameters was performed and the z-values were considered in the analyses (both genders included together because of the separately calculated z-values), in order to guarantee the comparability (according to previous studies [6]). The predicted APHV was directly used for analyses (separated for gender; without z-transformation), since it is independent from the actual chronological age of the individual.

The normal distribution of all parameters was tested using the Kolmogorov-Smirnov-Test. For evaluating differences in the single parameters (physical performance tests, anthropometric parameters, biological maturation) between ski racers and comparison group (separated for gender), Student’s T-Test or Mann-Whitney-U-Tests were used. For assessing the influence of the level of physical performance, the anthropometric parameters and the biological maturation on the RAE, univariate analyses of variance, and for non-normal distributed variables, parameter-free univariate analyses of variance (Kruskal-Wallis-H-Tests) were used (dependent variable: tested fitness ability, anthropometric parameters, biological maturation; independent variable: relative age quarter). The variance homogeneity was tested using the Levene-Test and for post-hoc-tests, those of Scheffé were used.

The level of significance was set at p<0.05. All calculations were performed using PASW Statistics V.21.0, and the effect size was assessed using G*Power 3.1.9.2.

## Results

### Relative age quarter distribution

The χ^2^-statistics showed a significant difference between the relative age quarter distribution of the ski racers compared to that of the comparison group, which showed a nearly equal distribution (Q1: 24.5%; Q2: 26.6%; Q3: 25.2%; Q4: 23.7%) among the four quarters (χ^2^(3, N = 282) = 12.52; p = 0.006; ω = 0.2). Significantly more ski racers were born in the first relative age quarter compared to the last quarter with a decreasing number of athletes from Q1 to Q4 (Q1: 33.3%; Q2: 25.2%; Q3: 20.6%; Q4: 20.9%). A significant difference was also shown between the relative age quarter distribution of the male ski racers compared to the male pupils (χ^2^(3, N = 155) = 13.85; p = 0.003; ω = 0.34) with an over-representation of athletes born in the first quarter (36.8%) and an under-representation of athletes born in Q4 (17.4%). A significant difference was present also between the distribution of the female ski racers and that of the female comparison group (χ^2^(3, N = 127) = 7.97; p = 0.047; ω = 0.26). The descriptive OR and the corresponding χ^2^ for each quarter for the total sample and according to gender are presented in Table 2.

**Table 2:** Descriptive OR across all relative age quarters for ski racers.

sample		Q1:Q2	Q1:Q3	Q1:Q4
total sample [n = 282]	χ^2^	14.12	19.94	15.92
	p value	<0.001	<0.001	<0.001
	OR [95% CI]	1.32 [1.02–1.72]	1.62 [1.22–2.15]	1.59 [1.20–2.11]
male [n = 155]	χ^2^	6.53	6.72	17.97
	p value	0.01	0.01	<0.001
	OR [95% CI]	1.50 [1.06–2.12]	1.72 [1.20–2.49]	2.11 [1.41–3.15]
female [n = 127]	χ^2^	0.82	1.87	0.01
	p value	0.364	0.171	0.98
	OR [95% CI]	1.12 [0.75–1.67]	1.48 [0.95–2.31]	1.16 [0.77–1.73]

OR = odds ratio; CI = confidence interval; χ^2^ = chi^2^ statistics; Q1-4 = relative age quarter 1–4

### Influence of the level of physical performance on RAE

In all physical performance tests the youth ski racers performed significantly better than the comparison group in both genders. Concerning the influence of the fitness variables on the RAE, no significant differences were shown between the ski racers and pupils born in the single relative age quarters. Hence, no significant influence of the level of physical performance on the RAE could be assessed. The results (z-values) are presented in Table 3.

**Table 3:** Comparison of physical performance characteristics of youth ski racers and comparison group by relative age quartile distribution and results of analyses of variance and T-tests or U-Tests.

	relative age quarter	ski racers [n = 53] (M ± SD)	comparison group [n = 86] (M ± SD)	differences—ski racers vs. comparison group
S3-Check-Test—stabilization index^~^	First (Q1)	0.10 ± 0.54	-0.03 ± 0.76	***
	Second (Q2)	-0.35 ± 0.70	0.07 ± 0.84	***
	Third (Q3)	0.03 ± 0.68	0.13 ± 1.20	***
	Fourth (Q4)	-0.73 ± 0.64	0.11 ± 0.91	***
	Total	-0.21 ± 0.69	0.05 ± 0.90	***
Jump Coordination Test—time in s^~^	First (Q1)	-0.53 ± 0.55	-0.06 ± 0.85	***
	Second (Q2)	-0.42 ± 0.52	-0.10 ± 0.69	***
	Third (Q3)	-0.23 ± 0.31	0.27 ± 1.28	***
	Fourth (Q4)	-0.74 ± 0.57	1.94 ± 3.75	***
	Total	-0.46 ± 0.51	1.51 ± 3.72	***
Counter Movement Jump—jumping height in cm	First (Q1)	0.42 ± 0.97	0.08 ± 0.78	***
	Second (Q2)	0.17 ± 0.37	-0.19 ± 0.90	***
	Third (Q3)	0.14 ± 0.37	0.00 ± 1.07	***
	Fourth (Q4)	0.64 ± 0.77	0.01 ± 0.94	***
	Total	0.31 ± 0.66	-0.03 ± 0.90	***
Drop Jump—reactive strength index	First (Q1)	0.61 ± 1.05	-0.01 ± 1.07	***
	Second (Q2)	0.10 ± 1.19	-0.14 ± 0.63	***
	Third (Q3)	0.25 ± 0.59	-1.19 ± 1.27	***
	Fourth (Q4)	0.73 ± 0.58	-0.53 ± 1.15	***
	Total	0.38 ± 0.95	-0.17 ± 1.02	***
unilateral leg press strength test—max. strength relative to body mass right leg in N/kg	First (Q1)	-0.14 ± 2.22	-0.10 ± 0.77	***
	Second (Q2)	0.29 ± 0.82	-0.35 ± 1.08	***
	Third (Q3)	0.22 ± 0.44	0.26 ± 1.22	***
	Fourth (Q4)	0.91 ± 0.67	-0.02 ± 0.65	***
	Total	0.27 ± 1.28	-0.09 ± 0.97	***
unilateral leg press strength test—max. strength relative to body mass left leg in N/kg	First (Q1)	-0.57 ± 2.95	-0.07 ± 0.80	***
	Second (Q2)	0.29 ± 0.53	-0.31 ± 1.19	***
	Third (Q3)	0.23 ± 0.26	0.25 ± 1.14	***
	Fourth (Q4)	1.05 ± 0.62	-0.02 ± 0.64	***
	Total	0.19 ± 1.61	-0.07 ± 0.99	***
Back-Check-Test—max. isometric flexion core strength relative to body mass in N/kg	First (Q1)	0.30 ± 0.52	-0.16 ± 0.89	***
	Second (Q2)	0.21 ± 1.20	0.15 ± 1.01	***
	Third (Q3)	-0.13 ± 0.40	-0.22 ± 0.99	***
	Fourth (Q4)	0.94 ± 0.51	-0.08 ± 0.78	***
	Total	0.28 ± 0.85	-0.07 ± 0.93	***
Back-Check-Test—max. isometric extension core strength relative to body mass in N/kg	First (Q1)	0.19 ± 0.61	-0.15 ± 0.90	***
	Second (Q2)	0.09 ± 1.30	-0.30 ± 1.34	***
	Third (Q3)	0.14 ± 0.34	0.21 ± 0.93	***
	Fourth (Q4)	0.87 ± 0.56	0.01 ± 0.72	***
	Total	0.26 ± 0.88	-0.10 ± 1.04	***

***p<0.001;

^~^lower values indicate better performance level;

for all physical performance tests the z-values were used for analyses; M = mean; SD = standard deviation; Q1-Q4 = relative age quarter 1–4

### Influence of anthropometric parameters on RAE

When comparing the ski racers and the comparison group in body mass and height, the male and female ski racers showed significantly higher values in body height (males: U(155,173) = 8533; p<0.001; females: U(127,240) = 12283; p = 0.002) and body mass (males: U(155,173) = 7752; p<0.001; females: U(127,240) = 13292; p = 0.044). In the case of comparing ski racers and pupils of the comparison group separately for relative age quarter, significant differences in body height and body mass were shown only for those born in Q1 and Q2, with significantly higher values for the ski racers.

Concerning the influence of body mass and body height on the RAE, significantly higher values in body height (χ^2^(3, N = 282) = 14.42; p = 0.002) and body mass (χ^2^(3, N = 282) = 7.82; p = 0.05) were shown for ski racers born in Q1 compared to those born in the other quarters. No significant differences in the anthropometric parameters were shown for the comparison group. Consequently, a significant influence of body mass and body height on the RAE could be shown only for youth ski racers. The results (z-values) are presented in Table 4.

**Table 4:** Comparison of anthropometric characteristics of youth ski racers and comparison group by relative age quartile distribution and results of analyses of variance and T-tests or U-Tests.

anthropo-metric parameter	relative age quarter	ski racers [282] (M ± SD)	comparison group [413] (M ± SD)	differences—ski racers vs. comparison group
body height in cm	First (Q1)	0.20 ± 0.86	-0.06 ± 0.06	***
	Second (Q2)	0.09 ± 0.81	-0.06 ± 0.07	***
	Third (Q3)	-0.08 ± 0.91	-0.05 ± 0.06	n.s.
	Fourth (Q4)	-0.29 ± 0.76	-0.04 ± 0.06	n.s.
	Total	0.01 ± 0.85	-0.05 ± 0.06	***
	Kruskal Wallis H-Test	χ^2^(3, N = 282) = 13.06; p = 0.005	n.s.	
body mass in kg	First (Q1)	0.14 ± 0.90	-0.37 ± 0.48	***
	Second (Q2)	0.14 ± 0.86	-0.31 ± 0.45	***
	Third (Q3)	-0.09 ± 0.89	-0.23 ± 0.37	n.s.
	Fourth (Q4)	-0.21 ± 0.70	-0.18 ± 0.31	n.s.
	Total	0.02 ± 0.86	-0.27 ± 0.42	***
	Kruskal Wallis H-Test	χ^2^(3, N = 282) = 9.07; p = 0.028	n.s.	

n.s.: not significant;

***p<0.001;

for all anthropometric characteristics, the z-values were used for analyses; M = mean; SD = standard deviation; χ^2^ = chi^2^-statistics; Q1-4 = relative age quarter 1–4

### Influence of biological maturation on RAE

No significant differences were assessed in the calculated APHV between youth ski racers and the comparison group (separated for gender). Moreover, no significant differences were shown in the APHV between the pupils born in the four relative age quarters, neither for the ski racers, nor for the comparison group. The results are presented in Table 5.

**Table 5:** Comparison of biological maturation characteristics of youth ski racers and comparison group by relative age quartile distribution and T-tests or U-Tests.

APHV in years	relative age quarter	ski racers [n = 282] (M ± SD)	comparison group [n = 413] (M ± SD)	differences—ski racers vs. comparison group
male	First (Q1)	13.72 ± 0.52	13.54 ± 0.64	n.s.
	Second (Q2)	13.74 ± 0.61	13.46 ± 0.48	n.s.
	Third (Q3)	13.76 ± 0.57	13.35 ± 0.38	n.s.
	Fourth (Q4)	13.84 ± 0.47	13.55 ± 0.45	n.s.
	Total	13.76 ± 0.54	13.48 ± 0.50	n.s.
female	First (Q1)	12.18 ± 0.60	12.05 ± 0.43	n.s.
	Second (Q2)	12.07 ± 0.57	11.90 ± 0.40	n.s.
	Third (Q3)	12.26 ± 0.56	11.91 ± 0.47	n.s.
	Fourth (Q4)	12.19 ± 0.53	11.85 ± 0.52	n.s.
	Total	12.17 ± 0.56	11.92 ± 0.46	n.s.

n.s.: not significant; APHV = age at peak height velocity; M = mean; SD = standard deviation; Q1-4 = relative age quarter 1–4

## Discussion

In Austria, ski boarding schools play an important role in the talent development system of alpine ski racing, as 90–95% of the members of the Austrian Ski Federation frequented such schools. As a result, the selection pressure in the entrance exams of these schools is high. Not surprisingly, in the present study a significant RAE with an over-representation of athletes born early in the selection year was present in this sample of youth ski racers who were recruited from ski boarding schools. This is in line with previous studies where a RAE was present in all categories of ski racing [6]. The descriptive OR of the current study revealed that the likelihood of being selected for frequenting a ski boarding school is 1.62 times higher for an athlete of the first relative age quarter than for one of the third quarter, and 1.59 times higher for a ski racer of Q1 compared to one of the last quarter.

In various sports, such as handball and basketball [37], RAEs often do not occur among female athletes. These and other authors hypothesize that a RAE does not exist in female contexts because the female variant of several sports is not particularly strength and power related; therefore, the maturation-related development lead is not as decisive [2]. Conversely, Romann and Fuchslocher [38,39] revealed a significant RAE among female soccer players. Further, Raschner et al. [9] found a significant RAE among the female participants of the 1^st^ YOG in 2012, and Müller et al. [6,17,19] reported a significant RAE among female youth ski racers attending ski boarding schools [17], female participants in the FIS Junior World Ski Championships [19] and female participants at the youngest levels of youth ski racing in Austria [6]. No significant RAE could be found among female World Cup ski racers. A stronger RAE was present among male athletes in the FIS World Cup and the FIS Junior World Ski Championships than among female athletes in those events [19]. Hence, a RAE is indeed present in female alpine ski racing; however, it is more pronounced among males. This finding is consistent with those of the present study, in which a RAE was present in both male and female youth ski racers. When comparing the effect sizes, a higher effect size was reported for the male athletes (ω = 0.34) compared to the female athletes (ω = 0.26). Additionally, the OR calculation revealed that the likelihood of frequenting a ski boarding school of a male athlete of Q1 compared to a male athlete of Q4 was higher (OR = 2.11 [1.41–3.15]) than that for a female athlete (OR = 1.16 [0.77–1.73]). The findings of the present study and of previous studies suggest that the RAE in alpine ski racing affects not only the male athletes, but also the female athletes; this result suggests that the female variant of this sport is also strength and power related and that the selection pressure is also high in the female context, as for example in Austria [17].

As previously mentioned, Wattie et al. [21] underline the importance of using a domain-specific model to explain the RAE in a specific sport meaning that the diverse interacting factors that influence participation should be investigated in each specific sport. The factors influencing participation and consequently causing a RAE can differ from sport to sport. Consequently, it is necessary to find the most important influential mechanisms on the RAE in alpine ski racing as well. This was the main goal of the present study because it has not been investigated before in the literature. An additional novelty of the present study and in RAE research was to include a comparison group of non-ski racers or non-athletes in order to be able to assess whether potential mechanisms influencing the RAE are generally valid or exist only among youth ski racers.

The results of the present study suggest that the level of physical performance had no significant influence on the RAE because there were no significant differences in single physical performance tests between athletes of the four relative age quarters. To date, only one study has investigated the influence of the level of physical performance on the RAE in alpine ski racing [17]. The results of that study are comparable to those of the present study, because no significant influence of the level of physical performance on the RAE was revealed. However, the ski racers were significantly better in all physical performance tests when compared to the group of non-athletes. This finding highlights the importance of a high level of physical fitness in youth ski racing, which has been documented in various [22–26]. Müller et al. [17] reported that entrance exams at ski boarding schools do not further intensify the RAE and that a substantial portion of the selection process has already been completed before the entrance exam into a ski boarding school takes place. As a consequence and based on the findings of the present study, it can be assumed that young alpine ski racers selected some time during the development system before entering a ski boarding school are physically homogenous, and therefore, they achieve the same level of physical performance, irrespective of relative age. The results also are comparable to the studies of Till et al. [7,27], in which no significant differences were found in the performance characteristics between players born in the single relative age quarters in UK Junior Rugby. They argued that coaches select similar archetypal athletes: if an athlete of relative age quarter 4 was to be selected, he required similar physical characteristics to an athlete born in Q1 [27]. Additionally, Votteler and Höner [40] compared the motor performance of youth soccer players selected for the German Football Association with normal motor development. They found accelerated motor performance values among relatively younger and retarded performance values among relatively older players compared to the expected normal development curve. [40] This result indicates that relatively younger soccer players are more likely to be selected if they have advanced physical characteristics. These findings are consistent with the results of the present study and lead to the assumption that youth ski racers born at the end of the selection year are more likely to be selected only if they have advanced physical characteristics.

The ski racers examined in the present study were significantly taller and heavier than the non-athletes. This finding is consistent with the previously described results of the study of Raschner et al. [11], in which a significant correlation could be found between alpine slalom racing performance in slalom and anthropometric characteristics. The pupils in the non-athlete comparison group did not significantly differ in height and weight, regardless of their relative age. This finding was surprising, because Votteler & Höner [40] reported that the weight and height of youth soccer players steadily increases from relative age quarter to relative age quarter. This was not the case in the comparison group of non-athletes in the present study, which may be due to the use of a z-transformation to guarantee comparability. However, the fact that ski racers of Q1 significantly differ in height and weight compared with other ski racers suggests that there are advantages to a higher level of anthropometric characteristics in alpine ski racing development. The significant influence of body mass and height on the RAE was only present among the ski racers; there was no significant difference among those in the comparison group. It can be hypothesized that as early as this young age (10–13 years), relatively older ski racers are more likely to be selected when they have advanced anthropometric characteristics, particularly when, as was the case in the present study, ski racers born in Q1 and Q2 had higher body mass and height compared to the group of non-athletes. In addition, the anthropometric characteristics seem to further influence the selection process in alpine ski racing, as the higher body height and body mass values of the athletes born in Q1 were only present in the ski racers. Raschner et al. [9] also found a significant influence of body mass and height on the RAE in male participants of the YOG 2012 with athletes born in the first quarter showing higher values compared to those born later. These results for male athletes are comparable to those of the present study, because Raschner et al. [9] also examined athletes of winter sport disciplines. However, they did not show any significant influence of anthropometric characteristics on the RAE for female athletes. Since alpine skiing is a sport that requires a high level of physical fitness and a sport with high demands on power, strength and body size [22], it is a sport in which a RAE is most likely to exist [15] and in which athletes with higher body mass and height have advantages [11]. Additionally, since many authors explain the over-representation of athletes born early in the selection year as a result of being chosen for their larger anthropometric dimensions, the influence of body mass and height on the RAE in alpine ski racing seems plausible. The results of the present study are not consistent with those of studies in soccer [29–30], which reported no significant influence of anthropometric characteristics on the RAE. However, the results are consistent with the findings of a study investigating the influence of anthropometric characteristics in winter sport disciplines at the YOG 2012 [9]. These findings led to the assumption that at least among males, anthropometric characteristics seem to influence the RAE in winter sport disciplines and particularly in alpine ski racing the influence is also present among females.

Anthropometric parameters are important for physical performance; therefore it could be assumed that, due to the fact that the ski racers of Q1 were significantly taller and heavier compared to the other ski racers, the relatively older ski racers also performed significantly better in the physical performance tests. However, this outcome was not observed in the present study. However, Votteler and Höner [40] reported that above-average scores in motor performance tests of relatively younger soccer players were not caused by advantages in physical development status. Hence, the advantages of the relatively older ski racers with respect to advanced anthropometric characteristics do not seem to influence the physical performance level; however, they do seem to influence alpine ski racing performance and consequently the selection process.

No significant differences in APHV, and consequently, in maturity status, were present between youth ski racers of the single relative age quarters. This finding indicates that all the athletes will reach their individual peak growth spurt at nearly the same age. These results are consistent with the findings of soccer studies [12,32], in which no significant differences were shown between players born in the single relative age quarters; the reported APHV was nearly the same. Deprez et al. [12,30] suggested that the smaller number of soccer players born later in the selection year have a better chance of being selected when they are advanced in biological maturation. Hence, they can counteract the RAE and the associated disadvantages if they enter puberty at an earlier age. Because taller and heavier athletes have advantages in alpine ski racing [11] and because body mass and height, which are both associated with maturation [13], influence the RAE in alpine skiing, it could be assumed that biological maturation would also influence the RAE. Because it was shown that selected athletes of the last relative age quarter show an equal or advanced biological maturation status compared to relatively older athletes [12], the missing difference in APHV between the ski racers of the single relative age quarters could be partly attributed to this reason. It seems that relatively younger ski racers have better chances of being selected when they are at similar or more advanced maturation levels, which Deprez et al. [12,30] suggested for youth soccer players. Additionally, Deprez et al. [12] divided their sample into three maturity groups (late, normal, early maturing) and compared the distribution of the three groups within the four relative age quarters. They demonstrated that within the first birth quarter late maturing athletes were overrepresented compared to early maturing athletes and within the last birth quarter it was the other way round; hence, they concluded that being born in the first birth quarter increases the chance of being selected for the elite level independent of the maturation status. On the other hand, players born in the last quarter may have increased chances of selection if they reach their peak height velocity at an earlier age. The same analysis was then additionally performed in the present study. However, similar results were not found, as the distribution of the three maturity groups did not differ significantly between the ski racers of the four relative age quarters.

It was hypothesized that youth ski racers would be more mature compared to pupils of the same age. Surprisingly, this could not be proven in the present study; the APHV of the ski racers did not differ significantly from that of the comparison group. However, the fact that youth ski racers are significantly taller and heavier compared to the group of non-athletes indicates that the combination of an older age and increased anthropometric characteristics appears to provide significant advantages in the talent selection process for young ski racers. Additionally, relatively younger ski racers can better counteract the disadvantages of the relatively younger age by having the same level of maturation as the relatively older athletes. The findings of the present study are therefore consistent with those of other studies of soccer and rugby, which reported that biological maturation had no significant influence on the RAE.

## Limitations

Due to logistical reasons, the physical performance tests could only be performed by a sub-sample of 139 pupils, who additionally could not be selected randomly. The authors recognize this point as a limitation of the study. However, the group of ski racers selected for the physical performance tests was compared with the rest of the total sample of ski racers with respect to height, weight and APHV. The same analyses were performed for the non-athlete pupils selected for physical performance tests and the rest of the comparison group. In both cases, there were no significant differences in height, weight or APHV between the study participants selected for the physical performance tests and those not selected, who were included in the analyses concerning anthropometric and maturational characteristics. Therefore, the participants selected for physical performance tests seem to be representative sub-samples.

## Conclusion

In conclusion, the results of the present study indicate that relatively younger youth ski racers can counteract the relative age disadvantage if they show the same level of physical fitness and maturity as the relatively older athletes, which is comparable to studies in soccer [12,32]. Additionally, the talent selection process in alpine ski racing seems to be influenced by anthropometric characteristics, as relatively older ski racers are taller and heavier compared to relatively younger ski racers and to non-athletes of the same age. Relatively older athletes have an increased likelihood of being selected when they have advanced anthropometric characteristics. Surprisingly, the youth ski racers did not show an advanced maturational status compared to the non-athletes, which was hypothesized due to the advantages of an advanced maturity status in alpine ski racing. This said, further research regarding the influence of the biological maturity on the RAE within the most talented youth ski racers seems important in order to appropriately change strategies in the talent selection process (e.g. including an estimation of biological maturity) to minimize the RAE in the future. Talent development systems have to consider anthropometric and maturational characteristics in order to contribute to more fairness.
